# Chronic musculoskeletal ankle disorders in Sri Lanka

**DOI:** 10.1186/s12891-017-1580-7

**Published:** 2017-05-25

**Authors:** Ishanka Weerasekara, Claire E. Hiller

**Affiliations:** 1 Department of Physiotherapy, Faculty of Allied Health Sciences, University of Peradeniya, Peradeniya, Sri Lanka; 2 Faculty of Health Sciences, The University of Sydney, Sydney, Australia

**Keywords:** Ankle sprain, Prevalence, Musculo-skeletal disorders, Chronic injuries, Ankle disabilities, Chronic sprains, Ankle injuries, Epidemiology, Rehabilitation

## Abstract

**Background:**

Musculoskeletal disorders of the lower extremities are commonly affected by chronicity and disability. One of the most commonly affected areas is the ankle. Epidemiological information is limited for chronic musculoskeletal ankle disorders in the general community, particularly in the developing world. This study aimed to determine the prevalence and impact of chronic musculoskeletal ankle disorders in the Sri Lankan community.

**Methods:**

A cross-sectional stratified random sample of people (*n* = 1000) aged 18 to 85 years in Sri Lanka was undertaken by questionnaire in the general community setting. Of those questionnaires, 827 participants provided data.

Point prevalence for no history of ankle injury or ankle disorders, history of ankle injuries without chronic ankle disorders, and chronic ankle disorders were obtained. Point prevalence of chronic musculoskeletal disorders and causes for chronicity was evaluated.

**Results:**

There were 448 (54.2%) participants with no ankle disorders, 164 (19.8%) with a history of ankle injury but no chronic disorders, and 215 (26.0%) with chronic ankle disorders.

The major component of chronic ankle disorders was musculoskeletal disorders (*n* = 113, 13.7% of the total sample), most of which were due to ankle injury (*n* = 80, 9.7% of the total). Sprains were responsible for 17.7% of the total ankle injuries. Arthritis was the other main cause for chronicity of ankle disorders with 4% of total participants (*n* = 33).

**Conclusions:**

Almost 14% of the Sri Lankan community was affected by chronic musculoskeletal ankle disorders. The majority were due to a previous ankle injury, and arthritis. Most people had to limit or change their physical activity because of the chronic ankle disorder. A very low utility of physiotherapy services was observed.

**Electronic supplementary material:**

The online version of this article (doi:10.1186/s12891-017-1580-7) contains supplementary material, which is available to authorized users.

## Background

Chronic musculoskeletal conditions are a global health problem [1, 2] and a major burden on individuals, affecting people’s families, the health system and society. Musculoskeletal conditions significantly affect the psychosocial status of the affected individuals and their families [3]. Musculoskeletal conditions are the most common cause of severe long- term pain, functional limitations and physical disability [3, 4]. Moreover, musculoskeletal conditions have been reported to be the most common cause of pain in the United Kingdom and internationally [5, 6]. Occurrence, chronicity and the associated disabilities of these conditions have a major impact on society resulting in problems in quality of life of the affected person [3]. Moreover, chronic musculoskeletal conditions are common medical causes of long term absence from work [3, 7] and a major reason for sick leave claims [3].

Musculoskeletal disorders of the lower extremities are commonly affected by chronicity and disability [1]. One of the most commonly affected areas is the ankle [8]. Garrick et al. reported that 14% of high school injuries were ankle injuries of which 85% were ankle sprains [9] and 72% of elite collegiate American football players had a history of foot and ankle injuries [10]. Within the general community incidence rates of ankle sprain were reported as 7 per 1000 in Denmark [11], 6.09 per 1000 in United Kingdom [12] and 2.15 per 1000 person-years in the United States [13].

Long term consequences of ankle sprain include pain, perceived ankle instability and recurrent sprain [2]. The impact on day to day life following ankle sprain has been reported to include returning to work with some impairment (15%) and inability to maintain any occupational activity (6%) [14]. Furthermore 72% of people with ongoing ankle disorders were unable to maintain their previous activity level [15].

Most of the information about long term consequences of ankle injury have been researched in specific groups, such as sport players [16] or people presenting to health services in the western world [13]. Studies reflecting the presence and impact of these consequences in the general community or the developing world, are scarce [2, 17]. Hiller et al. have shown there is a high prevalence of chronic ankle disorders which have a significant, adverse impact on health and quality of life in the Australian community [2]. While Kaur and Sinha evaluated the prevalence and service utilization of ankle sprains in players (non-specific) in Punjab, India, and found high recurrence rates of ankle sprains similar to western countries. They noted the importance of the availability of a physiotherapist at training and competition venues to complete rehabilitation and prevent recurrence [17].

The aim of this study was to determine the point prevalence of chronic musculoskeletal ankle disorders in the general community and investigate the impact of these disorders and health care use in Sri Lanka. Further, ﻿in the Sri Lankan community, people tend to depend on traditional treatments [18] or on self-management of these disorders. Therefore, conducting a survey of the general community was undertaken by the authors rather than within specific sport groups or people presenting to medical clinics.

## Methods

### Sampling

Sampling was undertaken based on the distribution of administrative units in Sri Lanka according to the data of 14^th^ National Census conducted in 2012. Altogether 9 provinces are divided into 25 districts and those districts have 331 District Secretariat (DS) divisions. These DS divisions consist of 14,021 Grama Niladari (GN) divisions.

**Fig. 1:**
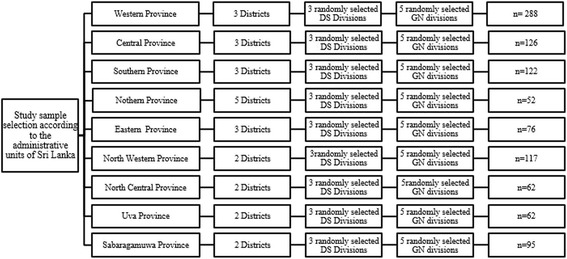
Planned study sample recruitment according to the administrative units of Sri Lanka

Three DS divisions from each District were randomly selected by the main investigators and 5 GN divisions were selected randomly from within the selected DS divisions. Interviewers were sent to those GN divisions to interview participants. Interviewers were instructed to randomly select a house to fill the first questionnaire of each GN division, and then collect data from every third house thereafter according to systematic selection. One volunteer participant from each house was selected for the study. Participants were informed that the study was on ankle injuries before they confirmed their volunteer participation (Fig. 1).

### Selection of the interviewers

With the permission of the Head of the Institution; volunteer data collectors were selected from the second, third and fourth year physiotherapy students of the Department of Physiotherapy, Faculty of Allied Health Sciences, University of Peradeniya. Applications were called for the students whose hometowns were in the randomly selected GN/DS divisions by the main investigators and from those who would like to volunteer to visit the selected GN/DS divisions. The students were given a special training tutorial regarding the study including: systematic selection of the house and participant from each GN/DS division, interviewing the participant, and completing the questionnaire. Interviewers were not given a specific definition of ‘musculoskeletal’ but as physiotherapy students had an adequate knowledge to discern from the participant description and history whether the disorder was of musculoskeletal origin.

### Procedure

The questionnaire from the study of Hiller et al. [2] was modified for use in this study (Additional file 1 Supplementary digital content 1) with the permission of the author. One thousand questionnaires were distributed according to the proportional allocation of the population of Sri Lanka. Selected interviewers collected data during a two month period from November 2014 to January 2015.

Participants were included in the study if they were aged 18 years to 85 years, and from either gender. The age criteria was pre-determined due to restrictions with parental permission (younger age) and ease of recruitment (older age).

### Data entry and data analysis

Descriptive statistics for the prevalence rates of chronic musculoskeletal ankle disorders, impact of the disorders and use of health care services were calculated. The total sample was classified into three subgroups: participants with no history of ankle injury and no chronic disorders, participants with a history of ankle injury but no chronic disorders, and participants with chronic ankle disorders. The last group was further divided into disorders of musculoskeletal and non- musculoskeletal origin. The chi- square test was used to investigate the differences between the three groups at difference age levels and for gender. Data were manually entered into a spreadsheet and analysed with IBM SPSS Statistics for Windows (Version 23.0 Armonk, NY: IBM Corp).

## Results

### Sample

**Fig. 2:**
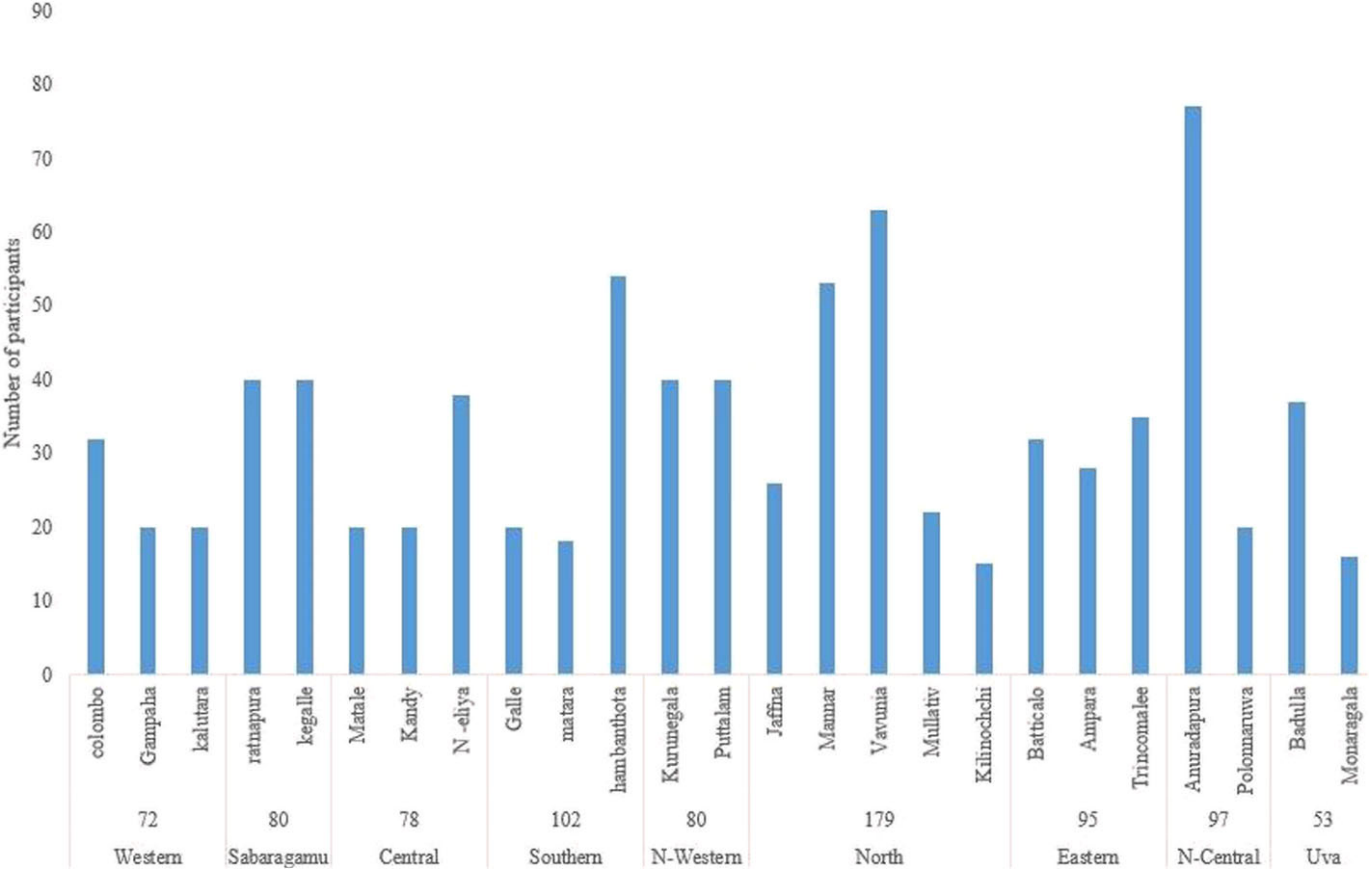
Recruited study sample according to the administrative units of Sri Lanka

One thousand questionnaires were distributed among the interviewers and 827 from all districts (Fig. 2) were returned by the interviewers during the time frame (return rate of 82.7%). Gender distribution of the total sample was 442 (53.4%) females to 385 (46.6%) males with mean age of 39.5 ± 15.5 years (mean ± SD).

A higher proportion of males (*n* = 87, 53.0%) than females (*n* = 77, 47.0%) had a history of ankle injury, but no ankle disorders. However, in the group with ankle disorders, there was a higher proportion of females (*n* = 118, 54.9%) than males (*n* = 97, 45.1%) (Table 1). However, these values were not statistically significant (Pearson chi square = 3.47, df =2, *p* = 0.176).

**Table 1:** Gender distribution of the participants

Category	Gender distribution		
	Total	Male % (*n*)	Female % (*n*)
Total study sample	827	46.6% (385)	53.4% (442)
No ankle injury, nor ankle problems	448	44.9% (201)	55.1% (247)
History of ankle injury, but no ankle problems	164	53.0% (87)	47.0% (77)
With chronic ankle problems	215	45.1% (97)	54.9% (118)

A high prevalence of ankle injuries was reported among 16–30 year olds without leading to chronicity (*n* = 69, 42.1%). Most chronic disorders were observed in the 46–60 years group (*n* = 73, 33.9%). There was statistically significant difference between the three groups across the age levels (Pearson chi square = 64.98, df =10, *p* < 0.0001).

### Prevalence of chronic ankle disorders

The prevalence of chronic ankle disorders was 26% (*n* = 215) and of these, the highest percentage were due to musculoskeletal disorders (52.6%, *n* = 113). Non musculoskeletal disorders were reported as 25.1% (*n* = 54) while 22.3% (*n* = 48) failed to respond. Foot ulcers were common among the non musculoskeletal disorders in the sample.

The majority of the participants with chronic ankle disorders reported the problem as lasting for nearly one year (*n* = 72, 33.5%). The prevalence decreased with time and only 5.6% (*n* = 12) had chronic problems lasting longer than 10 years (Additional file 1 Supplementary digital content 2).

With regard to the identified chronic disorders of musculo-skeletal origin (*n* = 113); 80 (70.8%) were due to ankle injuries and 33 (29.2%) were due to arthritis. The profile of ankle injuries included sprains 47.5% (*n* = 38), strains 15.0% (*n* = 12), fractures 22.5% (*n* = 18) and dislocations 5.0% (*n* = 4) with the remaining 10.0% (*n* = 8) due to other injuries. When considering the arthritic sample, 63.7% (*n* = 21) were due to osteoarthritis (OA) and 21.2% (*n* = 7) rheumatoid arthritis (RA), with the remainder unable to remember what type of arthritis they had (Table 2). However, 97% of arthritic cases were doctor diagnosed.

**Table 2:** Profile of the chronic musculoskeletal disorders

Origin of the disorder	Type of disorder	Cause of disorder	Percentage % (*n*)
Non- Musculoskeletal origin			25.1 (54)
Musculoskeletal origin			52.6 (113)
	due to ankle injuries		37.2 (80)
		sprains	17.7 (38)
		strains	5.6 (12)
		fractures	8.4 (18)
		dislocations	1.9 (4)
		other injuries	3.7 (8)
	due to arthritis		15.3 (33)
		osteoarthritis (OA)	9.8 (21)
		rheumatoid arthritis (RA)	3.3 (7)
		unknown	2.3 (5)
Not mentioned			22.3 (48)

### History of ankle disorders

Overall 379 people reported a history of ankle disorders (45.8%). Of these 164 had a history of ankle injury but no ongoing problems, while 215 had ongoing ankle problems Almost one third of the participants with a history of ankle disorders reported ongoing musculoskeletal problems (*n* = 113). Sprains were the most common injury (*n* = 98). Recurrence was reported for16.8% of musculoskeletal injuries.

### Impact of chronic musculoskeletal ankle disorders

Among the major complaints, pain was the most common (*n* = 142, 66.0%) and the majority of participants reported that the pain was moderate (92, 64.5%) and occurred often (59, 41.5%) Swelling was the next most common complaint (*n* = 54, 25.1%) followed by instability (*n* = 15, 7.0%) and weakness (*n* = 14, 6.5%) (Table 3).

**Table 3:** Major complains due to the chronic ankle disorder

Complain	Category		Percentage % (*n*)
Pain			66.0 (142)
	existence of the pain	persistent	23.9 (34)
		often	41.5 (59)
		occasional	34.5 (49)
	severity of the pain intensity	mild	12.7 (18)
		moderate	64.8 (92)
		severe	22.5 (32)
Swelling			25.1 (54)
Instability			7.0 (15)
Weakness			6.5 (14)

The majority of the sample with chronic ankle disorders had limited physical activities due to the ankle problem (70.6%, *n* = 152). Most participants had issues that affected walking and running (34.4%, *n* = 74), and activities of daily living (ADL) and house hold activities (5.6%, *n* = 12). ADLs were considered as five activities; movement in bed, transfers, dressing, personal hygiene, and feeding. Information on walking was collected separately. Standing, working, and sports were some other activities limited by the ankle problem (Table 4).

**Table 4:** Important physical activities limited due to chronic ankle disorder

Limited physical activity	Percentage % (*n*)
Walking and running	34.4 (74)
ADL and house hold activities	5.6 (12)
Standing	5.6 (12)
Working	2.3 (5)
Playing and sport	3.7 (8)
Other complains	6.5 (14)
None	12.6 (27)
Not mentioned	29.3 (63)

### Health care consultation during the course of chronic musculoskeletal ankle disorders

Two thirds of participants had sought health care in the past, and 34.9% of them (*n* = 75) had consulted a doctor. Traditional treatment was the second most visited health care 18.1% (43) among participants. However 7.9% (*n* = 17) self-treated using ice therapy, hot fomentation, bandage, pain killers or ointments (Additional file 1 Supplementary digital content 3).

Health care consultation during the past year showed that the practitioner most consulted was a doctor (*n* = 60. 27.9%) and 22.3% (*n* = 48) followed Traditional treatment. A physiotherapist was consulted by only 5.1% (*n* = 11) and Additional file 1 Supplementary digital content 4 describes the percentages of consultancy.

## Discussion

The point prevalence of chronic ankle disorders was almost 26% of the sample with the majority being of musculoskeletal origin. The most reported ongoing symptom was pain, in addition, and most people had limited their physical activities in some way. The prevalence and effects were similar to a western population with a few exceptions.

### Sample distribution

A high prevalence of ankle injuries were reported among 16–30 year olds without leading to chronicity (35.3%) which may be due to higher recovery rates at younger ages. Most chronic disorders were observed in the 46–60 years group (36.3%), again this may be due to the influence of age. Prevalence and severity of musculoskeletal conditions has been shown to increase with age [4] which supports the findings of this study.

More males had a history of ankle injury but no ongoing ankle problems while more females had chronic ankle problems. Though these findings were not statistically significant, they were in line with the conclusion of a systematic review conducted by Doherty et al., which found that females were at a higher risk of sustaining ankle sprains [7]. A similar gender distribution was observed in an Australian community [2].

### Prevalence of chronic musculoskeletal ankle disorders

The overall prevalence of chronic ankle disorders in the Sri Lankan study sample was lower than the Australian study sample [2]. In both studies the higher proportion of these disorders was of musculoskeletal origin, the majority due to ankle injuries and secondarily arthritic conditions. The most reported ankle injury in both communities was sprains and the most common arthritis was osteoarthritis.

Nearly one quarter of people in this Sri Lankan sample (*n* = 215) reported a chronic ankle disorder of which almost half of the participants (*n* = 113) had chronic disorders that were of musculoskeletal origin. The rate of 1 in 7 Sri Lankans having a chronic musculoskeletal ankle disorder was less than the Australian community where it affected 1 in 5 Australians [2]. One possibility for the higher prevalence of chronicity of the problem in the Australian community may be the difference in average age between two study samples. The Australian community was on average six years older and so had a longer time to sustain an injury. Across the total sample in this study, 4.6% of people were affected by ankle sprains which lead to chronicity. In a UK study, only 2.77% of the population had ankle sprains diagnosed at Accident and Emergency departments, however this may be an underestimation of the true prevalence because a proportion of patients with ankle sprains may not seek health care [13], reflecting patient’s thoughts that ankle sprains are minor injuries which do not require a health service. Possibly it is not stressed enough to the public that these injuries have preventable complications.

One significant difference between the Sri Lankan and Australian communities was the ankle injury prevalence. In Sri Lanka less than half the sample (43.2%) had a history of an ankle injury while in Australia it was nearly two thirds of the community (64.5%) [2]. This may be because of the lower level of involvement in sport and physical activity in the Sri Lankan community [19]. Participation in certain sports and exercise increases the risk of ankle injury [20] so an increased exposure to risky activities could explain the higher prevalence in Australia.

### Impact of chronic musculoskeletal ankle disorders

Pain was the most common chronic symptom, followed by swelling and instability. The burden of chronic pain is reported in several musculoskeletal conditions [3, 5] and it is supported by observations in this study on ankle disorders. Nearly half the people with pain experienced it often and more than two thirds rated the pain as moderate in severity. By comparison, even though pain was the major complaint in the Australian study, swelling was not a major complaint.

Most of the people had limited their physical activities due to the ankle disorder (70.6%), mainly walking and running in this study. The lack of effect on sporting activity is probably due to the lack of participation in sports in the Sri Lankan general community, (similar to many Asian countries) compared to western countries [21–23].

The most consulted health care practitioner was the Doctor, with traditional treatment also popular as a care provider. Traditional medicine caters to 60–70% of primary health care needs of the Sri Lankan rural population [18]. Though there is a declining trend in use of the traditional treatments, it is still popular for certain conditions including fractures and dislocations [24, 25]. Ayurveda physicians trained by ancestral teachers and institutionally trained practitioners deliver the traditional treatment to the public [25]. An external poultice made up of warm herbal paste is applied to the ankle joint for sprains, dislocations and fractures in the general practice of traditional medicine. However, there is limited evidence and information about herbs and therapies as they pass from generation to generation as secret remedies.

Reasons for the high reported percentage of ankle chronicity in the Sri Lankan community may be due to several reasons. First, ankle injuries like sprains are often neglected as they are not considered to be severe injuries, and therefore the proportion of people who seek health care is limited. Initially, poor following of the standard acute management; RICE protocol (rest-ice-compression-elevation) of injuries, especially after ankle sprains is frequently observed. Even though these were simple steps to follow, they may have ignored due to lack of awareness﻿. Second, rehabilitation via Physiotherapy is important and a combination of care utilising the Doctor and Physiotherapy was received by only 13 people. In the current Sri Lankan health system, a doctor usually refers for physiotherapy and rarely a patient consults a physiotherapist. The doctor may not refer the patient for physiotherapy rehabilitation, or there may be a lack of physiotherapy services. The patients may not be able to afford the cost or time for physiotherapy or other rehabilitation services. Future follow-up qualitative studies should be implemented to explore the potential reasons behind this limited exposure to physiotherapy and rehabilitation services in the Sri Lankan health system. Third, for a minority, social and lifestyle factors such as wearing slippers as opposed to sandals and covered shoes may also affect the chronicity of the problem.

### Implications

The prevalence of chronic musculoskeletal ankle disorders was high, as reported in other countries. Therefore these results can be used to stress to the general public and health care professionals, not to consider these problems a minor priority. Awareness programmes at school/club/society levels, may be effective in treating the initial injury and reducing chronicity in the Sri Lankan community. Not only awareness of rehabilitation services such as physiotherapy, but also the acute rehabilitation process including the RICE protocol, basic stretching and strengthening exercises, should be raised among the general public and health care professionals.

### Study limitations

**Fig. 3:**
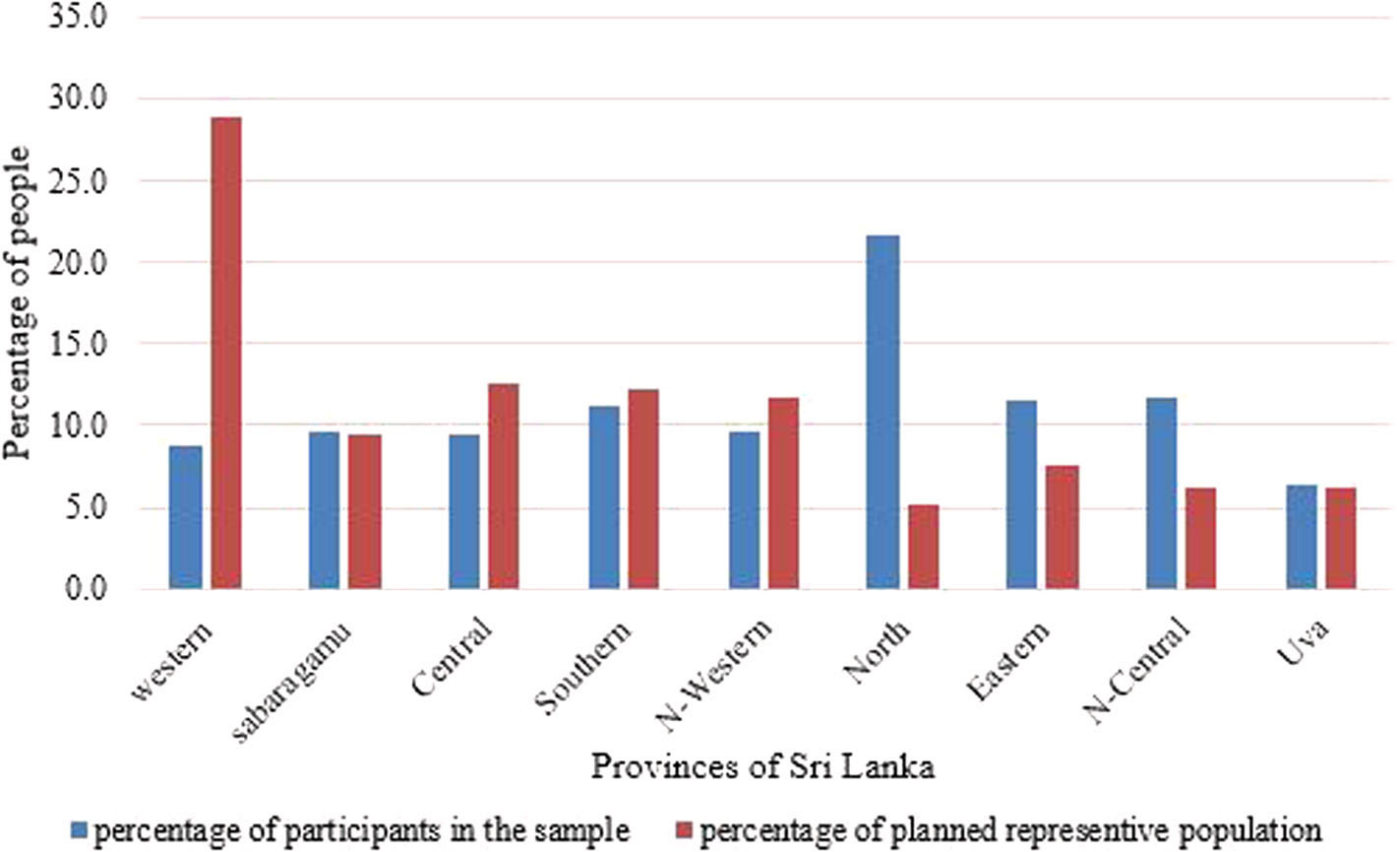
Comparison of actual and proposed population representation

There are few limitations to this study which may limit the generalisability of the results. First, due to time constraints, the planned proportionate distribution of questionnaires among districts was interrupted and the actual distribution of participants was less broad than planned (Fig. 3). Some of the interviewers failed to interview the allotted number of participants, so fewer questionnaires were returned resulting a response rate of 82.7%. Though the overall return rate was high, some responders did not give clear answers or did not answer some questions. The non-responses were included in the calculations so the reported prevalence rates are likely conservative. Further data was not weighted and thus may not truly represent the population. The understanding of level of musculoskeletal problems may differ from second year to fourth year students.

## Conclusions

This is the first survey to determine the prevalence of chronic ankle disorders in a developing country, Sri Lanka. Chronic musculoskeletal ankle disorders, affected 1 in 7 Sri Lankans. Pain, swelling and instability limited the physical activities of these people and implementing a way to receive proper health care consultancy is recommended. A similar picture emerges overall between developing and developed countries, with only the activities affected and types of health care practitioner consulted, varying. Chronic musculoskeletal ankle disorders are seen to be a global problem despite often being caused by a ‘simple’ sprain.

## Additional file

Additional file 1: Supplementary digital content 1: Questionnaire. This is the modified (with the permission of the author) version of the questionnaire originally from the study of Hiller et al., 2012. Supplementary digital content 2: Chronicity of the ankle problem of the sample. Duration of the problem, frequency and the percentage of the participants is described in this content. Supplementary digital content 3: Usual health care consultation. Percentage of the participants (out of 215) who consulted each health care provider is described here. Supplementary digital content 4: Health care consultation in the last year. Percentage of the participants (out of 215) who consulted each health care provider in the last year is described here. (DOCX 109 kb)

## Abbreviations

ADL: Activities of daily living
DS: District secretariat
GN: Grama niladari
OA: Osteoarthritis
RA: Rheumatoid arthritis
RICE: Rice-ice-compression-elevation
