# Supplementary material for: Chronic musculoskeletal ankle disorders in Sri Lanka
Source: BMC Musculoskelet Disord. 2017 May 25;18:219. doi: 10.1186/s12891-017-1580-7 (PMC5445495; doi:10.1186/s12891-017-1580-7)
Supplement: Additional file 1: — Supplementary digital content 1: Questionnaire. This is the modified (with the permission of the author) version of the questionnaire originally from the study of Hiller et al., 2012. Supplementary digital content 2: Chronicity of the ankle problem of the sample. Duration of the problem, frequency and the percentage of the participants is described in this content. Supplementary digital content 3: Usual health care consultation. Percentage of the participants (out of 215) who consulted each health care provider is described here. Supplementary digital content 4: Health care consultation in the last year. Percentage of the participants (out of 215) who consulted each health care provider in the last year is described here. (DOCX 109 kb) [file 12891_2017_1580_MOESM1_ESM.docx]

***Supplementary digital content 1 - Questionnaire***

Questionnaire: **Chronic Musculoskeletal Ankle Disorders in Sri Lanka**

Reference No: Date:

District:

Gender: Male- Female-

Birth Year:

1. Do you have any long-term problems with your ankles? That is, problems have lasted or are expected to last more than 6 months (eg, pain, weakness, swelling, arthritis and limping )

YES  go to 8

NO   go to 2

1. Have you ever injured your ankles? For example, sprains, strains, fractures (broken bones), dislocations.

YES  go to 3

NO   END

1. What injuries have you had?

Sprain of ligament

Strain of a muscle

Fracture (broken bones)

Dislocation

Other (Please specify) ______________________

1. Did you attend any of the following for treatment of your injury?

Doctor (general practitioner or specialist)

Government Hospital Private Hospital

Physiotherapist

Native treatments (Ayurvedic doctor)

Self- treatment (Heat/ Ice/Bandage/Exercise/Pain killers/Balm/Cream etc)

;Please specify_____________________

No treatments

Other (Please Specify) __________

1. How long ago?__________________________
2. Which of these best describes the activity that you were doing when you had your/latest injury?

Sporting activities

Leisure activities

Work-related activity

Attending school/college/university

Domestic activities

Other (Please Specify) __________

1. Do you have any problems NOW because of the injury?

YES  go to 14

NO   END

1. Is your ankle problem due to an injury to your ankle?

YES  go to 14

NO   go to 9

1. Is your ankle problem due to arthritis?

YES  go to 11

NO   go to 10

1. What is your ankle problem due to a musculoskeletal condition?

YES Go to 19

NO END

1. What type of ankle arthritis do you have, mention here if you know?

___________________________________________

1. Did a doctor tell you that you had arthritis?

YES

NO—please specify whom__________________

1. Is your arthritis due to a previous ankle injury?

YES  go to 14

NO   go to 16

1. Did the injury that is causing you problems occur in the

last month

6 months

nearly a year

1 to 5 years

5 to 10 years

more than 10 years?

1. Was the injury due to one of the following?

Sprain of ligament

Strain of a muscle

Fracture or broken bone

Dislocation

Other (Please Specify) __________

1. Did you attend any of the following for the treatment of your injury?

Doctor (general practitioner or specialist)

Government Hospital Private Hospital

Physiotherapist

Native treatments (Ayurvedic doctor)

Self- treatment (Heat/ Ice/Bandage/Exercise/ Pain killers/Balm/Cream etc)

; Please specify_____________________

No treatments

Other (Please Specify) __________

1. Is the injury a recurrence of a previous problem?_______________________
2. Do you suffer from any of the following due to your ankle problems?

Pain in or around your ankle

Ankle weakness

Ankle swelling

A feeling of your ankle giving way

A feeling of ankle instability

Any other ankle problem: Please specify___________________

If answered yes to pain go to 19.

(If not code as * go to Q21.)

1. Do you have pain in your ankle?

Always

Often

Occasionally

1. Is the pain

Severe

Moderate

Mild

1. How long have you had problems with your ankle?

Less than a year

1 to 5 years

5 to 10 years

More than 10 years

1. Does your ankle problem limit your physical activity?______________________

In what way?________________________________________________________________

1. What is the most important activity to you that you cannot do because of your ankle problem?

_____________________________________________________________________

1. Have you seek help for your ankle problem (not injury) in the past year from

Doctor (general practitioner or specialist)

Government Hospital Private Hospital

Native treatment (Ayurvedic doctor)

Physiotherapist

Pharmacist

Other (Please specify) __________

***Supplementary digital contentv2: Chronicity of the ankle problem of the sample***

| Duration (years) | Frequency | Percentage (%) |
| --- | --- | --- |
| 1 | 72 | 33.5 |
| 2-5 | 49 | 22.8 |
| 6-10 | 19 | 8.8 |
| More than 10 | 12 | 5.6 |
| Total | 215 | 100.0 |

***Supplementary Digital Content 3: Usual health care consultation***

| Health care provider | Consulted alone or as a combination of several treatment/s | Percentage %  (n out of 215) | |
| --- | --- | --- | --- |
| Doctor |  | 34.9% | (75) |
|  | alone | 21.9% | (47) |
|  | combined | 13.0% | (28) |
| Traditional treatment |  | 18.1% | (39) |
|  | alone | 9.8% | (21) |
|  | combined | 5.2% | (11) |
| Physiotherapist |  | 6.1% | (13) |
|  | alone | 0.0% | (0) |
|  | combined | 6.1% | (13) |
| Doctor +Physiotherapist |  | 6.1% | (13) |
|  | alone | 2.3% | (5) |
|  | combined | 3.7% | (8) |
| Other/ self -treatment |  | 9.3% | (20) |
|  | alone | 2.3% | (5) |
|  | combined | 7.0% | (15) |
| None |  | 0.5% | (1) |
| not mentioned |  | 51.6% | (111) |

***Supplementary Digital Content 4: Health care consultation in the last year***

| Health care provider | Consulted alone or as a combination of several treatment/s | Percentage % (n out of 215) | | |
| --- | --- | --- | --- | --- |
| Doctor |  | 27.9% | (60) | |
|  | Alone | 20.5% | (44) | |
|  | combined | 7.4% | (16) | |
| Traditional treatment |  | 22.3% | (48) | |
|  | alone | 15.8% | (34) | |
|  | combined | 6.5% | (14) | |
| Physiotherapist |  | 5.1% | (11) | |
|  | alone | 1.4% | (3) | |
|  | combined | 3.7% | (8) | |
| Doctor +Physiotherapist |  | 3.7% | (8) | |
|  | alone | 0.9% | (2) | |
|  | combined | 2.8% | (6) | |
| Other/self-treatment |  | 23.7% | (51) | |
|  | alone | 22.3% | (48) | |
|  | combined | 1.4% | (3) | |
| None |  | 1.9% | (4) |  |
| Not mentioned |  | 30.2% | (65) |  |
